# Self-assemblies of pluronic micelles in partitioning of anticancer drugs and effectiveness of this system towards target protein†

**DOI:** 10.1039/d1ra03770f

**Published:** 2021-06-22

**Authors:** Pooja Prasanthan, Nand Kishore

**Affiliations:** Department of Chemistry, Indian Institute of Technology Bombay Powai Mumbai 400 076 India nandk@chem.iitb.ac.in

## Abstract

Micelles formed by pluronic triblock copolymers are known to be a promising class of drug delivery vehicles. Quantitative mechanistic insights into the ability of pluronic micelles to improve the solubility of poorly water soluble drugs, encapsulation and delivery of hydrophilic drugs are not available. The current study evaluated the energetics of encapsulation of chemotherapeutic drugs gemcitabine, cytarabine, and hydroxyurea in pluronic F127 and F68 micelles. In addition, the interactions of the drugs released from pluronic micellar media with serum albumin, which is a major circulatory transport protein, and subsequent conformational changes have also been analyzed with the help of calorimetry and spectroscopy. All the drugs showed improved partitioning in F127 micelles, the extent of which slightly increased with temperature rise. Interestingly, drug–protein binding is enhanced upon delivery from pluronic micelles without affecting the conformational integrity of the protein. This study highlights the role of drug functionalities, hydrophobicity, and steric factors towards their partitioning in pluronic micelles. Such studies are important in understanding physicochemical aspects of drug encapsulation and release, and lead to establishing structure–property–energetics correlations for developing suitable nano-drug delivery vehicles.

## Abstract 2

Micelles formed by pluronic triblock copolymers are known to be a promising class of drug delivery vehicles.

## Graphical abstract

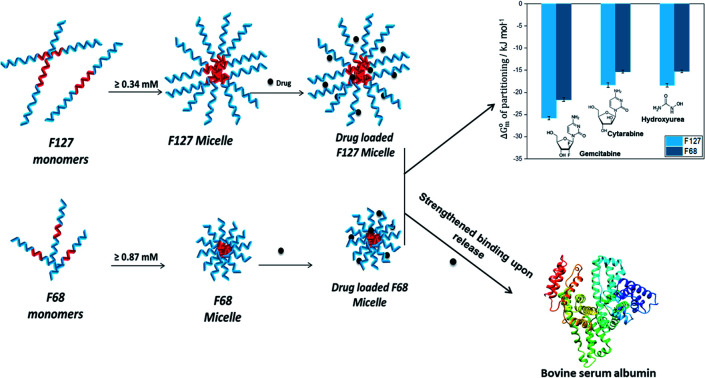

## 1 Introduction

Cancer is one of the prominent causes of death globally.^1^ Cancer treatment options include chemotherapy, and radiotherapy as well as surgical methods. Chemotherapeutic agents act by inhibiting the proliferation of cancer cells. Non-selective delivery of these agents can harm normal cells leading to unintended side effects.^2–4^ Therefore it is desirable to develop target-oriented drug delivery carriers for safe and efficient delivery for anticancer agents.

A wide variety of polymers have been extensively explored for the targeted delivery of therapeutic agents. Among the polymer based drug delivery systems, polymeric micelles have gained significant research interest because of their solubilization ability, high loading capacity, greater *in vivo* stability and therapeutic potential.^5–8^ Micelles formed by pluronic tri-block co-polymers are identified to be a promising class of drug carriers because of their bio-compatibility, bio-degradability, and greater ability to solubilize hydrophobic drugs.^9,10^ Pluronic polymers consist of hydrophilic poly (ethylene oxide) (PEO) units and hydrophobic poly (propylene oxide) (PPO) units organized as PEO_*x*_-PPO_*y*_-PEO_*x*_. These polymers self-aggregate in aqueous medium to form micelles with a PPO core bounded by PEO coronas.^11^ The average size of the pluronic micelles is of the order of 10–100 nanometers.^12^ Because of their lower toxicity and ability to form a transparent gel, pluronic polymers find wide applications as pharmaceutical excipients.^13,14^ The physicochemical properties of pluronic copolymers can be optimized by altering the molar ratio between PEO and PPO blocks.^15^ The higher kinetic and thermodynamic stability of pluronic micelles as compared to classic-surfactant micelles is due to the entangling of the polymer blockchain and combined molecular effects.^16^ Though pluronic micelles are extensively explored systems for the targeted delivery of poor water soluble anticancer agents,^17^ their ability to deliver hydrophilic anticancer drugs has not been addressed well. The therapeutic efficiency of hydrophilic drugs is often limited by enzymatic degradation, rapid clearance, and low cellular absorption. The pharmacokinetics of hydrophilic molecules can be enhanced by incorporating them in nanocarriers which allow prolonged release and targeted delivery.^18,19^

**Fig. 1:**
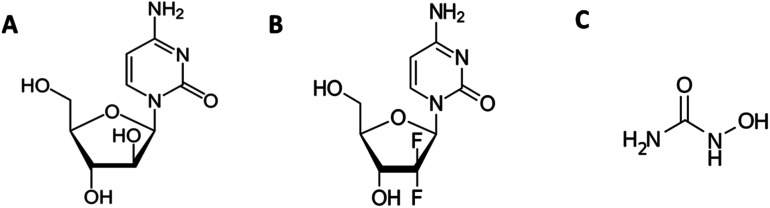
Chemical structures of (A) cytarabine, (B) gemcitabine and (C) hydroxyurea.

The current study focuses on the quantitative understanding of partition characteristics of anti-cancer drugs in pluronic F127 (PEO_100_-PPO_65_-PEO_100_) and F68 (PEO_76_-PPO_29_-PEO_76_) micelles and their interaction with protein upon release from micelles. Pluronic F127 and F68 are commonly used pluronic polymers for drug delivery applications. The encapsulation of cytarabine (Cyt), gemcitabine (Gem), or hydroxyurea (HU) into F127 and F68 micelles was quantitatively probed by using ultra-sensitive isothermal titration calorimetry. The chemical structures of these drugs and micelles are shown in Fig. 1. Cyt is a synthetic pyridine nucleoside analogue drug approved for the treatment of myeloid leukemia.^20^ Gem is also a nucleoside analogue that shows resemblance to cytarabine in structure and metabolism. It is primarily used in the treatment of small cell lung cancer and pancreatic cancer.^21,22^ Hydroxyurea is an antimetabolite which does not have any structural similarity with cytarabine or gemcitabine. It is used in the treatment of melanoma as well as leukemia.^23,24^ Bovine serum albumin is chosen as a model protein to study drug–protein interactions in the presence of these pluronic polymers. An in-depth quantitative understanding of drug partitioning in polymeric micelles in terms of thermodynamic signatures is helpful in providing guidelines for choosing suitable carriers for effective delivery of drugs. The design and development of efficient strategies for the targeted delivery of therapeutic agents are very important in drug discovery.

## 2 Materials and methods

### 2.1 Materials

Pluronic F127, hydroxyurea, and bovine serum albumin (BSA) of best available purity grade were obtained from Sigma Chemicals Company, USA. Pluronic F68 was purchased from Thermo Fisher Scientific and gemcitabine and cytarabine were procured from TCI Chemicals Pvt. Ltd. BSA solution was extensively dialyzed against 20 mM phosphate buffer for 12 hours at 4 °C with at least 3 changes of the latter. The concentration of the dialyzed protein was determined from the absorbance measurements by using an extinction coefficient, *E*^1%^_280_ = 6.8.^25^

### 2.2 Isothermal titration calorimetry (ITC)

The ITC experiments were carried out by using Nano ITC from TA Instruments. The volumes of cell and syringe of ITC are 170 μl and 50 μl, respectively. A total of 25 injections, each of 2 μl were added sequentially in to sample cell by using a computer-controlled syringe with 300 s time interval between consecutive injections. Nano Analyze software provided by TA Instruments was used to analyze ITC data.

F127 and F68 solutions were titrated into degassed water at various temperatures to determine their critical micelle concentration (CMC) at corresponding temperatures. The syringe was filled with drug solutions and titrated against surfactant solution taken in cell for partitioning studies. A concentration much higher than the CMC of pluronic polymers taken in sample cell (1 mM for F127 and 3 mM for F68) to retain polymers in micellar form throughout the partitioning studies. The interactions between drug and protein upon release from pluronic micelles were studied by titrating drug partitioned in F127 or F68 micelles at optimized concentrations against BSA. Dilution corrections were executed in this case as well.

### 2.3 Differential scanning calorimetry (DSC)

Thermal denaturation studies of BSA were done on Nano DSC (TA instruments) which has a cell volume of 300 μl. The amount of protein in all the experiments was maintained at 3 mg ml^−1^. DSC runs were performed at 1 K min^−1^ scan rate over a temperature range of 25–95 °C. The values of transition temperature and calorimetric enthalpy of unfolding of BSA under different conditions were determined by analyzing baseline corrected DSC profiles using Nano Analyze software.

### 2.4 Circular dichroism spectroscopy (CD)

The CD experiments were done on a Jasco-800 spectrophotometer. The far UV CD spectra were recorded using a cuvette of path length 0.2 cm with a protein concentration of 5 μM. The path length and concentration used for near UV CD measurements were 1 cm and 15 μM, respectively. Each reported spectra is an average of three measurements recorded at 100 nm s^−1^ scan rate. The molar ellipticity [*θ*] was determined by using equation given below.1
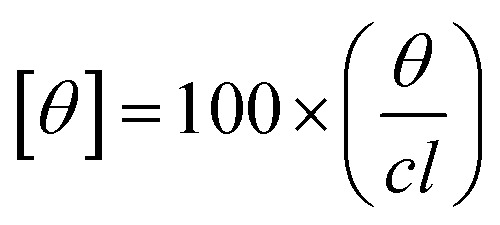


Here, *θ* is the measured ellipticity, *c* represents the protein concentration mol dm^−3^ and *l* is the cell path length in centimeters.

### 2.5 Fluorescence spectroscopy

A Cary Eclipse spectrophotometer was used to perform steady-state fluorescence measurements at 298.15 K. The excitation and emission slit width were fixed at 5 nm. The sample solutions were excited at 295 nm to selectively excite tryptophan residues of BSA and emission spectra were recorded from 310–500 nm. BSA concentration was kept at 2 μM and the concentration of the drug was varied from 0.5 μM to 1 mM. The fluorescence emission spectrum of the drug was also recorded as control experiments.

## 3 Results and discussion

### 3.1 Determination of critical micelle concentration of F127 and F68

**Fig. 2:**
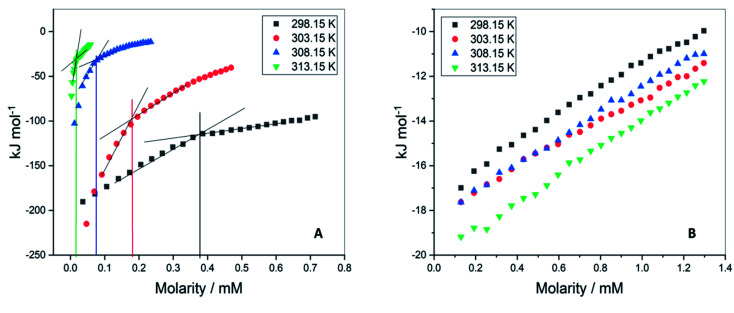
Representative ITC profiles for the titration of (A) F127 (B) F68 into water at 298.15 K, 303.15 K, 308.15 K, and 313.15 K.

The values of critical micelle concentration (CMC) of F68 and F127 in aqueous solution at different temperatures were determined using ITC. The inflection points of the titration curves are chosen as the value of CMC (Fig. 2). This model assumes the existence of equilibrium between free surfactant monomers and micelles at CMC.^26,27^ The concentration of F127 taken in the syringe was optimized at each temperature. The CMC of F127 at 298.15 K is determined to be (0.34 ± 0.01) mM which is in agreement with earlier reported range of CMC values (Table 1). The shape of the ITC profile depends on various factors such as aggregation number, temperature, surfactant concentration, the shape of the micelles and desolvation effects.^28–30^ The value of CMC showed a decrease from (0.34 ± 0.01) mM to (0.02 ± 0.01) mM with temperature rise from 298.15 to 313.15 K (Table 1). This suggests that an increase in temperature promotes micellization of F127. The rise in temperature leads to dehydration of PEO units thereby increases the hydrophobic character of the polymer chain.^31^ Thus these molecules can assemble into micelles at a relatively lower concentration at higher temperatures.

**Table:** Critical micelle concentration values of F127 at different temperatures

Temperature (K)	CMC (mM)		
	ITC studies	From literature	
298.15	0.34 ± 0.01	0.56 (ref. 36)	Fluorescence
		0.8 (ref. 37)	Surface tension
		0.5 (ref. 38)	Fluorescence
		0.01 (ref. 39)	Ultrasonic velocity
		0.02 (ref. 40)	Light scattering
		0.65 (ref. 41)	Solubility study
303.15	0.17 ± 0.02	0.079 (ref. 38)	Fluorescence
		0.008 (ref. 39)	Light scattering
308.15	0.06 ± 0.01	0.019 (ref. 38)	Fluorescence
		0.01 (ref. 39)	Light scattering
313.15	0.02 ± 0.01	0.006 (ref. 38)	Fluorescence
		0.005 (ref. 39)	Light scattering

The ITC profiles of F68 titration in water did not show a clear inflection point (Fig. 1B) making determination of CMC difficult by this method. The small deviation in data points only suggests the CMC of F68 to be in the range of 0.6–1 mM. The reported CMC values of F68 range from 0.4–1 mM.^32–34^ The CMC of F68 is found to be slightly higher than that for F127 in aqueous solution. F68 contains fewer hydrophobic, PPO units in comparison to F127. The HLB ratio (hydrophilic to lipophilic ratio) of F127 and F68 are 22 and 29,^35^ respectively. The greater hydrophobicity of F127 compared to F68 can be accounted for the smaller CMC value of the former.

### 3.2 Partitioning of drugs in F127 micelles: isothermal titration calorimetry studies

**Fig. 3:**
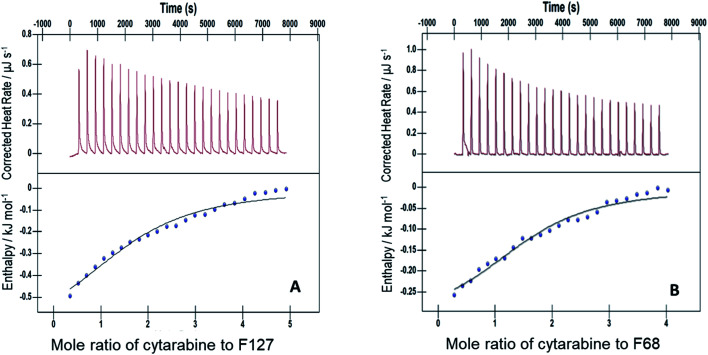
Representative ITC profiles of titration of cytarabine against (A) 1.1 mM F127 (B) 3 mM F68 at pH 7.4 and 298.15 K.

The interactions of Cyt, Gem, or HU with F127 micelles were studied in temperature range 298.15–313.15 K. The concentration of F127 in the cell of ITC was significantly higher than its CMC to maintain it in the micellar form even after addition of 25 injections. Representative ITC profiles for the titration of Cyt with F127 and F68 solution at 298.15 K are shown in Fig. 3.

The partitioning constant of Cyt in F127 micelles is found to be of the order of 10^3^ which showed a slight increase with increase in temperature (Table 2). The value of *K* varied from (1.6 ± 0.1) × 10^3^ M^−1^ at 298.15 K to (4.8 ± 0.2) × 10^3^ M^−1^ at 303.15 K. The value of standard molar enthalpy change 
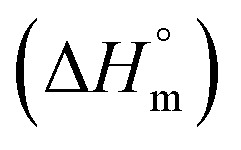
 associated with partitioning is weakly negative. The value of standard molar entropy change 
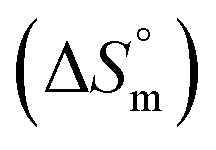
 is positive and showed an increase from (58.1 ± 1.2) to (69.3 ± 1.4) J mol^−1^ K^−1^ with temperature rise from 298.15–313.15 K. The observed enhancement in entropy can be attributed to desolvation of drug as well as micellar surface upon association. These results suggest that the partitioning of Cyt in F127 micelles is predominantly driven by positive entropy contributions. Insignificant enthalpy changes accompanying the partitioning process suggest compensation of heat effects due to polar interactions between Cyt and F127 with heat of desolvation of drug and micellar surface. Cyt has one –NH_2_ (amine group) and three –OH (hydroxyl groups), that can undergo polar interactions with hydrophilic –CH_2_–CH_2_–O– (PEO) units of pluronic F127.

The values of *K*, 
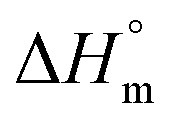
, 
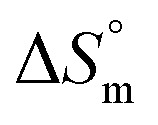
, and 
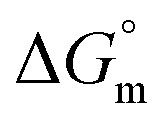
 associated with partitioning of gemcitabine, cytarabine or hydroxyurea in F127 and F68 micellar media at various temperatures

**Table:** 

	F127			
	*K* (M^−1^)	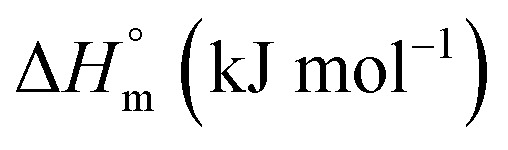	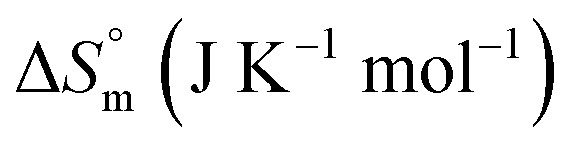	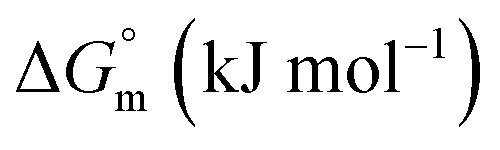
**Cytarabine**				
298.15	(1.6 ± 0.1) × 10^3^	−(1.0 ± 0.1)	(58.1 ± 1.2)	−(18.3 ± 0.4)
303.15	(2.9 ± 0.1) × 10^3^	−(0.9 ± 0.1)	(63.2 ± 1.3)	−(20.1 ± 0.4)
308.15	(3.7 ± 0.1) × 10^3^	−(0.7 ± 0.1)	(66.1 ± 1.3)	−(21.1 ± 0.4)
313.15	(4.8 ± 0.2) × 10^3^	−(0.4 ± 0.1)	(69.3 ± 1.4)	−(22.1 ± 0.4)

**Gemcitabine**				
298.15	(3.3 ± 0.7) × 10^4^	(1.3 ± 0.2)	91.2 ± 1.8	−(25.8 ± 0.5)
303.15	(4.0 ± 0.1) × 10^4^	(1.5 ± 0.3)	92.8 ± 1.3	−(27.6 ± 0.4)
308.15	(6.0 ± 1.0) × 10^4^	(1.6 ± 0.3)	96.9 ± 1.9	−(28.2 ± 0.5)
313.15	(1.0 ± 0.2) × 10^5^	(1.9 ± 0.3)	101.9 ± 2.0	−(30.1 ± 0.6)

**Hydroxyurea**				
298.15	(1.7 ± 0.3) × 10^3^	−(1.1 ± 0.2)	58.1 ± 1.0	−(18.4 ± 0.4)
303.15	(2.7 ± 0.5) × 10^3^	−(1.2 ± 0.2)	61.9 ± 1.2	−(19.9 ± 0.4)
308.15	(4.5 ± 0.9) × 10^3^	−(1.4 ± 0.3)	65.5 ± 1.3	−(21.6 ± 0.4)
313.15	(7.8 ± 1.6) × 10^3^	−(1.7 ± 0.3)	69.2 ± 1.4	−(23.3 ± 0.5)

**Table:** 

	F68			
	*K* (M^−1^)	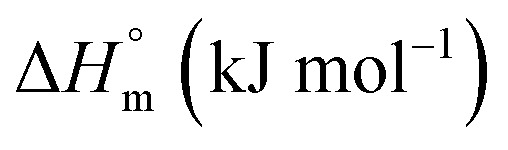	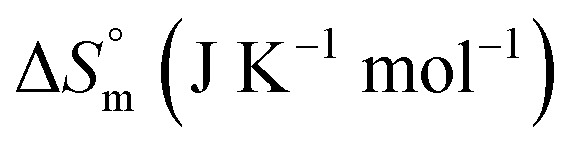	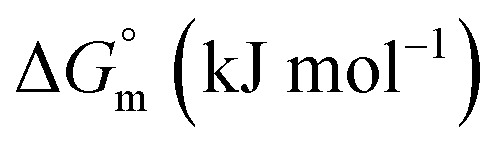
**Cytarabine**				
298.15	(4.8 ± 0.1) × 10^2^	−(0.4 ± 0.01)	50.2 ± 1.0	−(15.3 ± 0.3)
303.15	(5.7 ± 0.1) × 10^2^	−(0.3 ± 0.01)	51.8 ± 1.0	−(16.0 ± 0.3)
308.15	(8.3 ± 0.2) × 10^2^	−(0.3 ± 0.05)	55.0 ± 1.1	−(17.2 ± 0.3)
313.15	(9.3 ± 0.2) × 10^2^	−(0.3 ± 0.06)	56.0 ± 1.1	−(17.8 ± 0.4)

**Gemcitabine**				
298.15	(6.2 ± 0.1) × 10^3^	19.1 ± 0.38	136.5 ± 2.7	−(21.6 ± 0.4)
303.15	(7.8 ± 0.2) × 10^3^	13.5 ± 0.27	119.1 ± 2.4	−(22.6 ± 0.5)
308.15	(8.7 ± 0.1) × 10^3^	8.7 ± 0.17	101.7 ± 2.0	−(22.6 ± 0.4)
313.15	(2.1 ± 0.6 × 10^3^	2.1 ± 0.04	83.2 ± 1.5	−(24.0 ± 0.4)

**Hydroxyurea**				
298.15	(4.7 ± 0.1) × 10^2^	−(0.4 ± 0.1)	49.8 ± 0.9	−(15.2 ± 0.3)
303.15	(5.2 ± 0.9) × 10^2^	−(0.4 ± 0.2)	50.8 ± 1.0	−(15.3 ± 0.3)
308.15	(7.2 ± 0.4) × 10^2^	−(0.4 ± 0.1)	53.4 ± 1.1	−(16.3 ± 0.3)
313.15	(7.9 ± 0.5) × 10^2^	−(0.4 ± 0.1)	54.2 ± 1.1	−(17.4 ± 0.3)

The partitioning constant of Gem in F127 micelles is determined to be 10 folds higher than that of Cyt. The value of partitioning constant rises steadily with an increase in temperature (Table 2). The observed temperature-dependent strengthening of partitioning can be due to minor loss of structural rigidity of F127 micelles with the increase in temperature which allows comparatively more accommodation of Gem molecules. Here also the partitioning is entropy driven with 
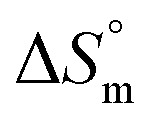
 values from (91.2 ± 1.8) J K^−1^ mol^−1^ to (101.9 ± 2.0) J K^−1^ mol^−1^ with temperature rise from 298.15 K to 313.15 K. Gem has significant hydrophilic content (two –OH groups, one –NH_2_, and two F groups) which can form polar interactions with hydrophilic PEO units of F127 micelles. The unfavorable enthalpy changes found here can be attributed to desolvation penalty. Here also, the positive enthalpy changes are compensated by entropic effects due to desolvation, thus driving the process towards spontaneity resulting in negative Gibbs free energy changes.

Gem displayed a 10 fold stronger partitioning in F127 micelles relative to Cyt. For both these drugs, partitioning is associated with favorable entropy change with pluronic micelles. Although both these drugs have significant hydrophilic content, the weakly positive value of 
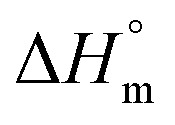
 for partitioning of Gem into F127 suggests stronger desolvation penalty of the two strongly hydrated F groups.^42^ The highest positive value of 
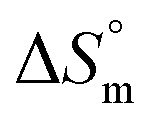
 for Gem also indicated a greater extent of desolvation of the latter in comparison to Cyt.

The values of *K*, 
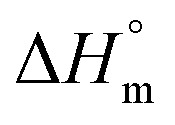
 and 
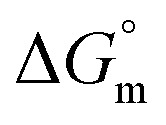
 accompanying the encapsulation of HU in F127 micelles are (1.7 ± 0.3) × 10^3^ M^−1^, −(1.1 ± 0.2) kJ mol^−1^ and −(18.4 ± 0.4) kJ mol^−1^, respectively at 298.15 K. In this case also weakly exothermic value of 
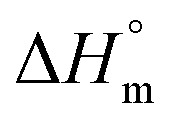
 and positive 
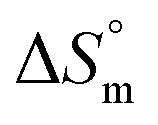
 implies that the partitioning process is primarily driven by entropy contributions. HU is a highly hydrophilic drug that can form polar interactions with hydrophilic PEO groups of F127. The thermodynamic quantities obtained for partitioning hydroxyurea in F127 micelles are comparable to that for Cyt.

### 3.3 Partitioning of drugs in F68 micelles

Cyt displayed 10 fold weaker partitioning in F68 micelles compared to that in F127 micelles. The partitioning constant of Cyt in F127 and F68 micelles are of the order of 10^3^ and 10^2^, respectively. The value of 
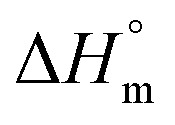
 is found to be weakly exothermic and entropic contributions are positive (Table 2).

The ITC profile for the titration of 25 mM Gem against 3 mM F68 is shown in Fig. S1.† The value of *K* for Gem in F68 micelles is equal to (6.2 ± 0.1) × 10^3^ M^−1^ at 298.15 K and does not show significant variations with rise in temperature. The value of 
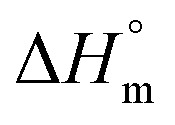
 is endothermic and 
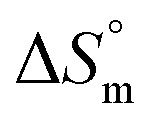
 is positive for the partitioning. Similar to Cyt and Gem, HU also displayed relatively weaker partitioning in F68 micelles compared to that in F127 micelles. The value of *K* associated with interaction of HU with F68 micelles is of the order of 10^2^. Thermodynamic parameters obtained from model fitting indicate that the interaction of HU with F68 micelles is weakly exothermic with positive entropy contributions (Table 2). The values of 
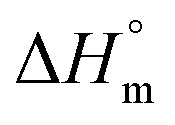
 and 
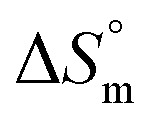
 are −(0.4 ± 0.1) kJ mol^−1^ and (49.7 ± 0.9) J mol^−1^ K^−1^, respectively at 298.15 K. Similar to that observed with F127 micelles, here also partitioning of all three drugs is entropically driven signifying the role of desolvation process.

Gem exhibited 10 folds stronger partitioning compared to the other two drugs in F127 as well as F68 micelles. The enthalpy of interaction between Gem and F127 or F68 micelles are observed to be endothermic while that for Cyt and HU is weakly exothermic in all the cases. The higher block chain length of F127 micelles compared to F68 micelles might be responsible for the relatively stronger partitioning of all these drugs in F127 micelles. The thermodynamic parameters associated with the interaction of HU and Cyt with F127 or F68 micelles are comparable even though HU is the smallest among all the drugs being studied.

### 3.4 Isothermal titration calorimetry of interactions of encapsulated Cyt, Gem and HU with BSA upon release

Isothermal titration calorimetry was employed to study the binding of drugs with protein upon release from micelles of F127 or F68. Many surfactants are known to bind to protein and hence modify interactions between drug and protein. Therefore it is important to investigate the interaction of drugs encapsulated in micellar media with protein and to understand if drug binding is affected due to components of the micelles. Upon addition of drug incorporated micellar solution into sample cell, micelles open up as a result of extensive dilution and lead to release of drugs. The binding of thus released drug molecules with BSA was analyzed by using ITC.

#### 3.4.1 Cyt–BSA interaction upon release from F127 or F68 micelles

**Fig. 4:**
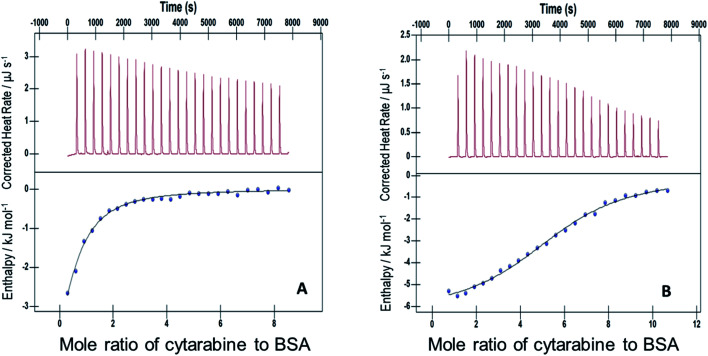
ITC profiles for the binding between cytarabine and BSA with (A) F127 and (B) F68 at 298.15 K.

Fig. 4 shows ITC profiles for the interaction between Cyt and BSA when delivered from F127 micelles. The value of *K* associated is (2.4 ± 0.1) × 10^4^ M^−1^ accompanied with exothermic enthalpy changes at 298.15 K (see Table 3). Exothermic enthalpy changes suggest predominance of electrostatic interactions between Cyt and BSA in the presence of F127. The positive value of 
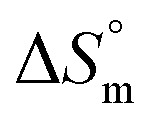
 is possibly due to desolvation effects such as desolvation of water molecules around protein and drug upon interaction. Cyt displayed a moderate affinity towards BSA with favorable enthalpy and entropy contributions when delivered from F68. Cyt–BSA interaction upon delivery from F68 micelles is associated with *K* = (2.0 ± 0.2) × 10^4^ M^−1^, 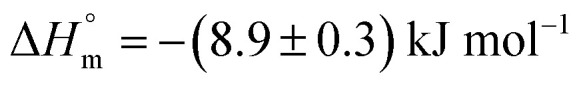 and (52.7 ± 1.3) J mol^−1^ K^−1^ at 298.15 K. The enthalpy changes became slightly more exothermic and entropy changes became less positive with F68 relative to that observed with F127. This can be attributed to the relatively greater hydrophobicity of F127.

The values of *K*, 
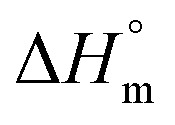
, 
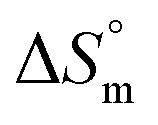
 and 
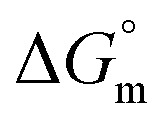
 associated with interaction of gemcitabine, cytarabine and hydroxyurea with BSA in the presence of F127 or F68 at pH 7.4 and 298.15 K

**Table:** 

F127	Cytarabine	Gemcitabine	Hydroxyurea
*K* (M^−1^)	(2.42 ± 0.12) × 10^4^	(6.53 ± 0.33) × 10^4^	(3.55 ± 0.18) × 10^4^
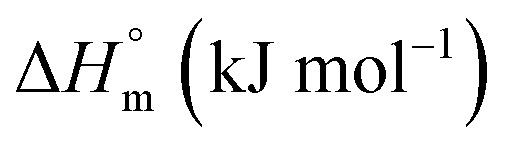	−6.28 ± 0.31	2.46 ± 0.12	3.53 ± 0.13
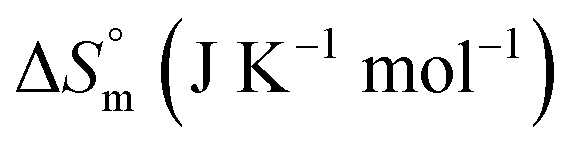	62.86 ± 3.14	100.40 ± 2.02	98.95 ± 4.95
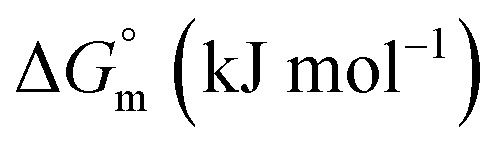	−25.02 ± 1.25	−(27.48 ± 0.14)	−(25.97 ± 1.30)

**Table:** 

F68	Cytarabine	Gemcitabine	Hydroxyurea
*K* (M^−1^)	(2.01 ± 0.18) × 10^4^	(8.50 ± 0.43) × 10^4^	(4.32 ± 0.21) × 10^4^
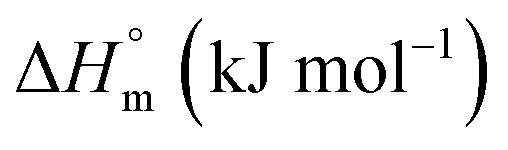	−(8.90 ± 0.28	0.76 ± 0.03	3.39 ± 0.17
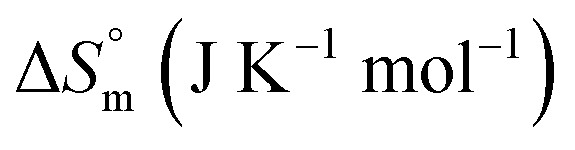	(52.71 ± 1.26)	96.92 ± 1.80	100.10 ± 2.01
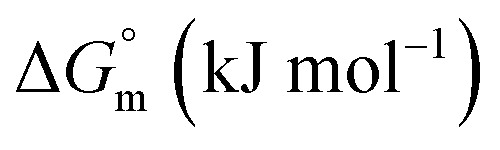	−(24.61 ± 1.20)	−(28.14 ± 1.61)	−(26.45 ± 2.1)

The ITC profile of the titration of Cyt with BSA in the absence of any additives is shown in Fig. S3.† The absence of specific binding profile observed implies no binding or predominantly entropically driven binding of Cyt and BSA with negligible enthalpy contribution. The observed enhancement in the binding affinity of Cyt towards BSA in the presence of pluronic F127 or F68 might be either due to the modulation of binding sites of protein or opening up on new binding sites on protein as a result of interaction with pluronic polymers.

#### 3.4.2 Gem–BSA interaction upon release from F127 or F68 micelles

Gem released from F127 micelles also displayed moderate binding affinity towards BSA alike Cyt with *K* of the order of 10^4^. The enthalpy changes associated with binding are determined to be slightly endothermic 
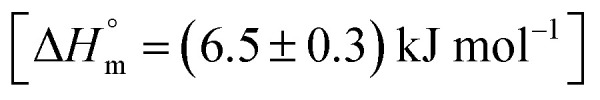
 and the value of entropy change is positive 
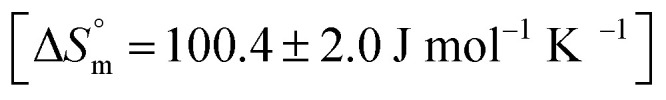
.

The thermodynamic signatures associated with the titration of Gem incorporated in F68 micelles with BSA are analogous to those observed in F127 micelles (see Table 3). The value of binding constant, standard molar enthalpy change and standard molar entropy change accompanied with the binding of Gem with BSA when delivered from F68 micelles are (8.5 ± 0.4) × 10^4^ M^−1^, (0.8 ± 0.1) kJ mol^−1^ and (96.9 ± 1.8) J K^−1^ mol^−1^, respectively. The interaction of Gem with BSA in the presence of F127 or F68 is found to be predominantly driven by favorable entropy contributions due to the release of water molecules upon drug desolvation.

Similar to Cyt, Gem also did not show specific binding interactions with BSA in the absence of any additives. The increased binding affinity of Gem to protein upon release from F127 or F68 micelles also suggested modified interaction between these drugs and BSA with pluronic polymers. The enthalpy changes associated became endothermic and entropy changes enhanced for binding of Gem with BSA compared to Cyt–BSA interaction. This suggests improved hydrophobic interactions of Gem with BSA compared to Cyt. The molecular structure of Gem differs from Cyt by substitution of fluorine at 2′ position. Fluorine substitution is responsible for the enhanced hydrophobic nature of Gem. Relatively large and positive entropy contributions to Gem–BSA interactions are also due to greater liberation of ordered water around hydrophobic parts of the drug due to desolvation.

#### 3.4.3 HU–BSA interaction upon relase from F127 or F68 micelles

The value of the binding constant associated with interactions of HU with BSA in the presence of F127 is (3.6 ± 0.2) × 10^4^ M^−1^. The endothermic enthalpy change along with positive entropy change (Table 3) indicates that the desolvation and solvent reorganization effects play a major role in the binding process. The interaction of HU encapsulated in F68 micelles with BSA showed a moderate binding affinity with *K*= (4.3 ± 0.2) × 10^4^ M^−1^. The binding is weakly endothermic and has positive entropy changes and. Alike Cyt and Gem, HU also did not bind to native BSA and showed a moderate affinity towards BSA in the presence of F127 or F68. The results suggest that the binding between HU and BSA when released from F127 or F68 micelles is predominantly driven by favorable entropy contributions from solvent reorganization effects.

#### 3.4.4 Interaction of F127 and F68 with BSA

**Fig. 5:**
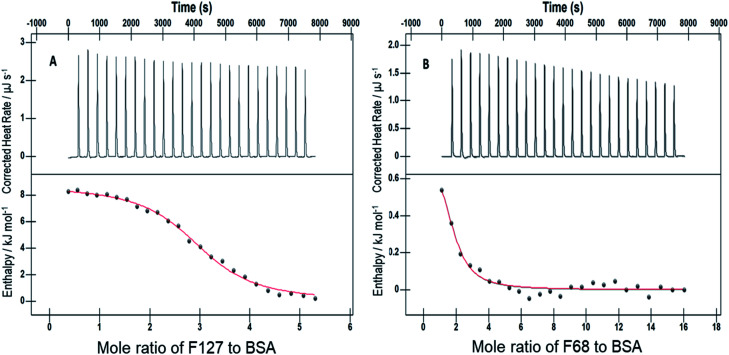
ITC profiles of the interaction of 1.1 mM F127 (A) and 3 mM F68 (B) with 0.06 mM of BSA at 298.15 K.

The strengthened binding of Cyt, Gem, and HU with BSA when delivered from F127 or F68 could be due to alteration of binding site(s) on protein as a result of interaction with polymers. Hence further experiments were carried out to understand binding of F127 or F68 with BSA. The ITC profiles for the binding of F68 or F127 with 0.06 mM BSA are shown in Fig. 5.

The accompanying thermodynamic parameters suggest moderate binding affinity between F127 or F68 and BSA with *K* of the order of 10^4^. The enthalpy of interactions between these polymers and BSA is found to be endothermic with favorable entropy changes suggesting the predominance of hydrophobic interactions (see Table 4). The hydrophobic chains of F127 or F68 can interact with non-polar patches on the BSA surface. The greater hydrophobic chain length of F127 can be accounted for the relatively more endothermic enthalpy contributions and more favorable entropy contributions associated with BSA–F127 binding compared to that for F68–BSA interaction. The results suggest that interactions between F127 or F68 and BSA play an important role in the modulation of binding of drug with the protein.

**Table:** The values of *K*, 
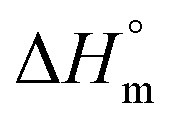
, 
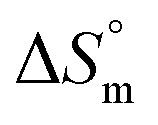
 and 
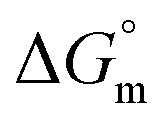
 associated with binding between F127 or F68 and BSA at pH 7.4 and 298.15 K

	F127	F68
*K* (M^−1^)	(1.50 ± 0.10) × 10^4^	(6.11 ± 0.31) × 10^4^
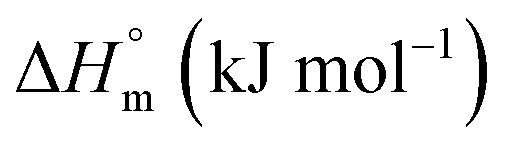	8.71 ± 0.22	0.82 ± 0.13
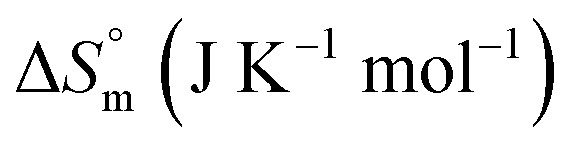	128.10 ± 1.13	94.41 ± 1.42
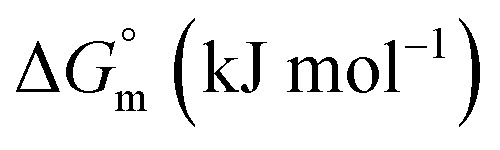	−29.50 ± 1.12	−27.30 ± 1.44

### 3.5 Fluorescence spectroscopy: interactions of encapsulated Cyt, Gem and HU with BSA upon release

Intrinsic fluorescence of BSA was monitored as a function of varying concentrations of drugs at 298.15 K. The fluorescence emission profile of protein at different concentrations of Cyt, Gem or HU ranging from 0 to 1 mM both in the absence and presence of pluronic F127 and F68 are shown in Fig. S4.†

A decrease in fluorescence intensity is observed with increasing concentration of the drugs. The quenching data was described by the Stern–Volmer equation.2
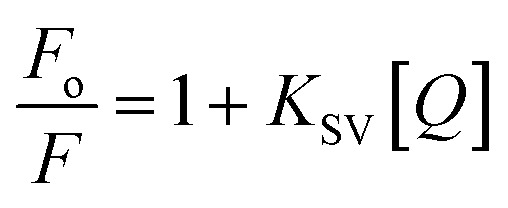


Here *F*_o_ and *F* represent the fluorescence intensities in the absence and presence of quencher, [*Q*] is the concentration of the quencher and *K*_SV_ is the Stern–Volmer quenching constant.

Cyt displayed greater extent of quenching followed by Gem and HU. The value of association constant, *K*_a_ stoichiometry of binding, *n* of interaction between drug and protein were evaluated by using the following equation.3
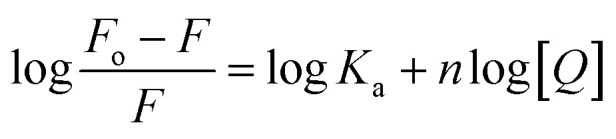


**Fig. 6:**
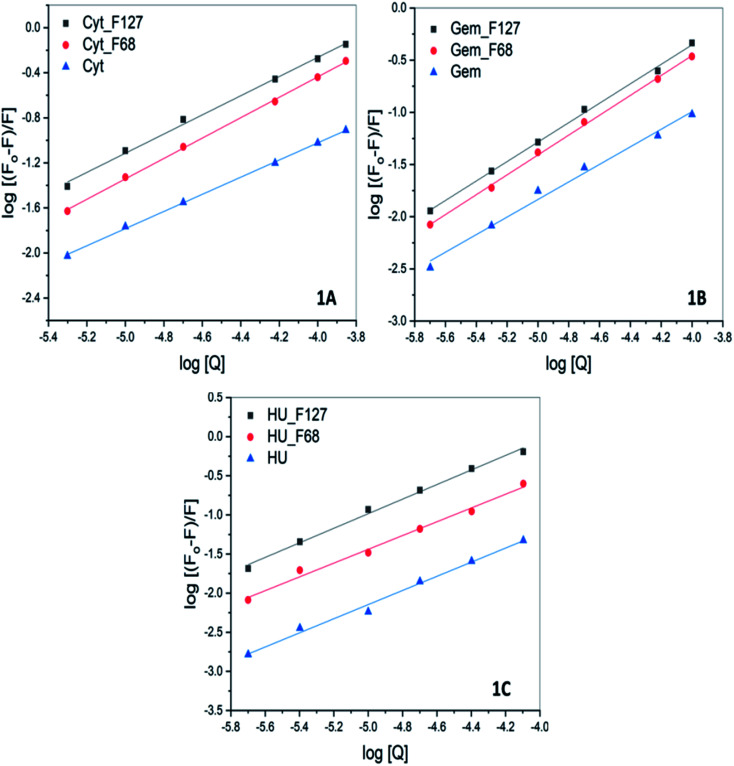
The log plot of fluorescence of BSA in presence of (A) cytarabine (B) gemcitabine (C) hydroxyurea in the absence and presence of pluronic F127 and F68 at pH 7.4 and 298.15 K. The plots corresponding to quenching by drugs in the presence of pluronics were corrected for contributions from quenching by only F127 or F68.

The values of *K*_a_ and *n* determined from the intercept and slope of the plot of 
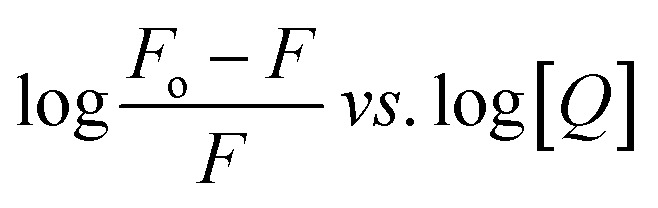
 (Fig. 6) are summarized in Table 5.

**Table:** The value of Stern–Volmer quenching constant (*K*_SV_) and association constant (*K*_a_) associated with interaction of cytarabine, gemcitabine, and hydroxyurea with BSA in the presence of F127 or F68 at pH 7.4 and 298.15 K

Drug		*K*_SV_ (M^−1^)	*K*_a_ (M^−1^)
Cytarabine	F127	(4.9 ± 0.1) × 10^3^	(1.4 ± 0.1) × 10^3^
	F68	(3.5 ± 0.3) × 10^3^	(1.5 ± 0.1) × 10^3^
	None	(8.4 ± 0.2) × 10^2^	(1.1 ± 0.1) × 10^2^
Gemcitabine	F127	(4.5 ± 0.2) × 10^3^	(1.5 ± 0.3) × 10^3^
	F68	(3.4 ± 0.2) × 10^3^	(2.2 ± 0.1) × 10^3^
	None	(9.0 ± 0.4) × 10^2^	(2.4 ± 0.2) × 10^2^
Hydroxyurea	F127	(7.9 ± 0.5) × 10^3^	(4.7 ± 0.2) × 10^3^
	F68	(3.0 ± 0.1) × 10^3^	(1.1 ± 0.3) × 10^3^
	None	(5.7 ± 0.2) × 10^2^	(2.3 ± 0.4) × 10^2^

The quenching studies suggested weak interactions between Cyt, Gem, or HU and BSA with association constants of the order of 10^2^. However, ITC studies suggested no specific binding interactions between these drugs and BSA. The discrepancy between ITC and fluorescence results indicates that the binding interactions between these drugs and BSA are predominately driven by favorable entropy contributions. This could be a major reason for the absence of heat effects in ITC profiles.

All these drugs showed a relatively greater extent of quenching when released from pluronic F127 and F68 micelles. The value of *K*_a_ associated with Cyt–BSA interaction is determined to be (9.9 ± 0.1) × 10^2^ M^−1^. While in the presence of F127 and F68 polymers the value of *K*_a_ increased to (6.4 ± 0.1) × 10^3^ M^−1^ and (1.2 ± 0.1) × 10^3^ M^−1^, respectively. Similarly, the value of *K*_a_ of interactions of Gem or HU also showed one order increase in the presence of micellar components (Table 5). The results imply improved binding affinity of all the drugs towards protein when delivered from F127 or F68 micelles. The ITC studies of the binding of encapsulated drugs with protein also suggested enhanced binding affinity in the presence of micellar components with a binding constant of the order of 10^4^.

#### 3.5.1 Circular dichroism spectroscopy: effect of pluronic F127 or F68 on conformation of protein

**Fig. 7:**
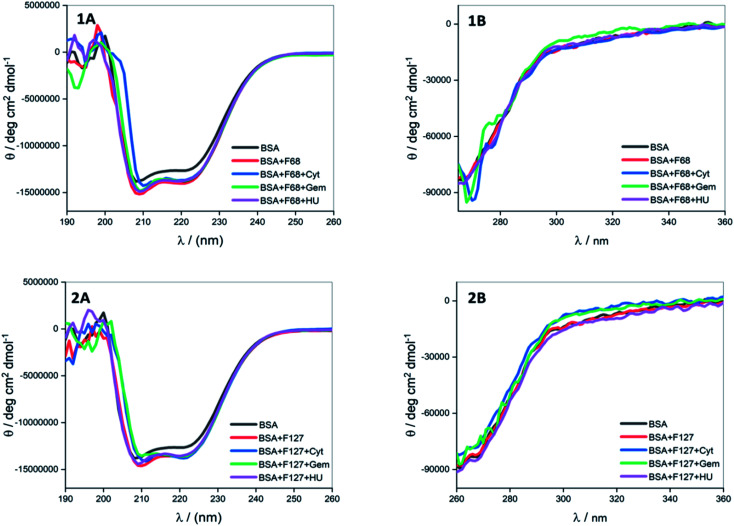
Far (A) and near (B) UV CD spectra of BSA in the presence of cytarabine, gemcitabine and hydroxyurea when delivered from (1) F68 and (2) F127 at 298.15 K.

The conformational changes in BSA due to the presence of these surfactants reflect on their impact on the integrity of protein binding sites. The concentrations of these polymers in CD experiments were chosen based on their final concentration in protein in the ITC experiments. It is seen in Fig. 7 that 0.7 mM F68 does not lead to a significant change in the tertiary structure of the protein while the secondary structure displayed a slight strengthening. Similar observations are made in the presence of 0.2 mM F127. The secondary structure is marginally stabilized and the tertiary structure is intact in the presence of F127 also. These results imply that both F127 and F68 do not significantly modify the protein binding sites under the experimental conditions used here.

#### 3.5.2 Effect of gemcitabine, cytarabine, and hydroxyurea on the conformation of BSA in presence of pluronic polymers

Native BSA did not show appreciable stabilization of secondary and tertiary structure in the presence of either of these drugs. The secondary and tertiary conformations of BSA did not show major variations in the combined presence of F68 or F127 and these drugs (Fig. 7). The conformation of BSA remained intact in the presence of Cyt, Gem, and HU with 0.2 mM F127 also. These results suggest that BSA can retain its conformation when these drugs are delivered from F127 or F68 micelles and the components of the micelles do not have any adverse effect on the protein stability. The CD experiments confirm that delivery of Cyt, Gem, and HU to BSA from F127 or F68 micelles retain the conformation of the protein under employed conditions.

### 3.6 Differential scanning calorimetry

Quantitative analysis of thermal properties of protein upon drug binding and effect of drug carriers is essential to evaluate the therapeutic potential of the latter. To assess thermal stability of BSA, DSC studies were carried in presence of drug, F127 or F68 individually and in the combined presence of drug and polymers.

#### 3.6.1 Thermal stability of BSA in the presence of F127 or F68

**Fig. 8:**
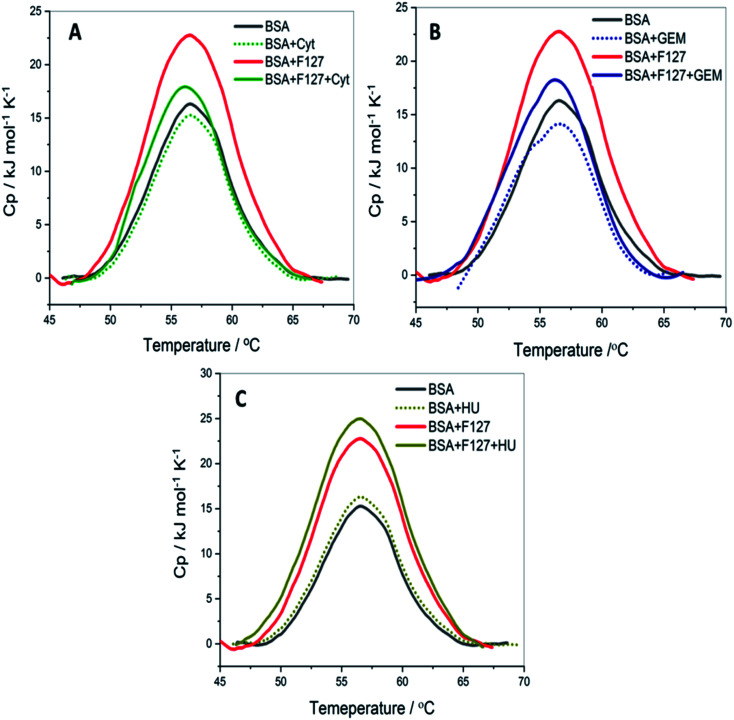
The DSC profiles of 3 mg ml^−1^ BSA with and without of F127 and (A) cytarabine (B) gemcitabine (C) hydroxyurea at pH 7.4.

The thermal unfolding of BSA is observed to be irreversible in the absence and presence of additives. DSC thermogram of native BSA showed a single endothermic transition with *T*_m_ = (55.1 ± 0.1) °C and enthalpy of unfolding, Δ_cal_*H*_m_ = 366 ± 3 kJ mol^−1^ (Fig. 8) these are in accordance with earlier reported values.^43^

0.2 mM F127 unfolds BSA at *T*_m_ = (56.1 ± 0.3) °C with a gain in enthalpy of ΔΔ_cal_*H*_m_ = 46 ± 4 kJ mol^−1^. This observation is supported by CD results in which a minor reinforcement of the secondary structure of BSA by F127 is identified. Protein stabilization by smaller concentrations of surfactants has been described earlier.^44,45^ The absence of significant structural changes in protein by F127 indicates that the integrity of BSA binding pockets is retained with the latter.

The DSC thermogram of BSA displayed a sharper transition with higher enthalpy of unfolding in the presence of 0.7 mM F68. The curve was deconvoluted in two endothermic transitions (see Fig. S5†). The value of *T*_m_ and Δ_cal_*H*_m_ associated with the transition I are (52.4 ± 0.3)°C and (162 ± 3) kJ mol^−1^, respectively. While that for transition II are (56.7 ± 0.6) °C and (331 ± 2) kJ mol^−1^, respectively. In order to gain more insights into this greater enthalpy gain, the DSC profile of F68 micellar solution alone was recorded. It is observed that F68 displays a weak transition with Δ_cal_*H*_m_ = (70.4 ± 2.1) kJ mol^−1^ and *T*_m_ = (58.8 ± 1.2) °C. This suggests structural reorganization of F68 monomers which require energy at higher temperatures. This endotherm corresponds to micelle formation. The difference in thermal behavior of BSA in the presence of F68 can be accounted for structural modifications of protein in the rearranged phase of the former at higher temperature. CD spectra exhibit no significant variations in conformation of protein with an equivalent concentration of F68 at 298 K. This indicates that temperature plays an important role in the interaction between BSA and the components of F68 micelles.

The results imply that pluronic polymers F127 or F68 micelles do not destabilize the protein. The absence of major variations in the thermal profile of BSA by components of F127 micelles suggests retention of the conformational integrity of the protein. Whereas the lack of conformational changes observed from CD studies and altered thermal profile of BSA in the presence of F68 surfactant suggested temperature dependent conformational changes of the protein by the latter.

#### 3.6.2 Thermal unfolding of BSA in the combined presence of F127 and drug

The impact of binding of the drug on the thermal behavior of protein when released from F127 micelle media was investigated. The thermal denaturation of (3 mg ml^−1^ BSA + 0.2 mM F127) was monitored in the presence 0.2 mM Cyt, 0.3 mM Gem or 0.7 mM HU. The DSC thermograms of BSA in the presence of only drug and the combined presence of drug and F127 are shown in Fig. 8. The values of *T*_m_ and Δ_cal_*H*_m_ associated with thermal unfolding are tabulated in Table 6.

**Table:** The thermodynamic parameters associated with thermal transitions of 3 mg ml^−1^ BSA in the presence of F127 in the presence of drugs at pH 7.4

Sample	*T*_m_ (°C)	Δ_cal_*H*_m_ (kJ mol^−1^)
BSA	55.6 ± 0.1	366 ± 3
BSA + F127	56.1 ± 0.3	412 ± 2
BSA + cytarabine	55.2 ± 0.1	363 ± 1
BSA + cytarabine + F127	55.9 ± 0.5	380 ± 2
BSA + gemcitabine	55.8 ± 0.1	362 ± 2
BSA + gemcitabine + F127	56.1 ± 0.2	390 ± 1
BSA + hydroxyurea	55.8 ± 0.1	367 ± 1
BSA + hydroxyurea + F127	56.1 ± 0.1	416 ± 2

The thermal unfolding parameters indicate that the stability of BSA is not appreciably affected by the presence of Cyt. This implies weaker binding between Cyt and BSA which is consistent with the ITC results. Whereas in the combined presence of F127 and Cyt, the enthalpy of unfolding of protein showed a marginal increase of (14.3 ± 2.1) kJ mol^−1^ compared to native BSA. However, no significant variations are observed in the *T*_m_ values. The small enhancement in the value of Δ_cal_*H*_m_ in the combined presence of Cyt and F127 suggests minor structural stabilization of BSA. The extent of increase is found to be smaller compared to that observed in the presence of only F127. These results suggest a slight alteration in the mode of binding between protein and Cyt by F127.

The thermal stability of BSA did not show major deviations with Gem analogous compared to that observed for Cyt. The value of melting temperature remained the same and the enthalpy of unfolding slightly increased by Gem when released from F127 micelles. The value of Δ_cal_*H*_m_ in the presence of Gem alone and in the combined presence of (Gem + F127) are (362 ± 2) kJ mol^−1^ and (390 ± 1) kJ mol^−1^, respectively. The relatively higher value of Δ_cal_*H*_m_ BSA in the presence of F127 alone compared to that in the presence of (Gem + F127) further suggests modifications of the binding interactions between Gem and BSA by F127.

The DSC thermograms of BSA with HU and (HU + F127) are shown in Fig. 8C. The extent of increase in enthalpy of unfolding with (0.7 mM HU + 0.2 mM F127) is higher than that in the presence of (0.2 mM Cyt + 0.2 mM F127) or (0.3 mM Gem + 0.2 mM F127). The value of Δ_cal_*H*_m_ increased by (50 ± 3) kJ mol^−1^ when HU is released from F127 micelles. These results indicate a relatively larger extent of variation of HU–BSA interaction by F127 with no change in *T*_m_ value. This can be attributed to the variation in the binding mode of HU with BSA compared to Gem or HU due to its distinct structure.

Overall, DSC results suggest minor structural stabilization of BSA in the presence of Cyt, Gem, or HU when delivered from F127. This is in accordance with the enhancement of binding affinity of drugs towards protein from micellar delivery.

### 3.7 Mechanistic aspects of partitioning

Quantitative understanding of the partition process is essential in the design of efficient micelle based drug formulations. In this work, we have investigated the partitioning of hydrophilic anticancer drugs Gem, Cyt, and HU in PEO-PPO-PEO triblock copolymeric micelles. All these drugs exhibited weak to moderate partitioning in pluronic F127 and F68 micellar media.

HU is a small hydrophilic drug consisting of one carboxyl group, one amino group, one imine group, and one hydroxyl group. It is capable of forming polar interactions with hydrated hydrophilic PEO corona of pluronic block copolymers. The order of partitioning constant of HU in F127 and F68 micelles are 10^3^ and 10^2^, respectively. Cyt is a synthetic nucleoside (deoxycytidine) analogue in which cytosine is attached to arabinofuranose sugar. It is bulky and contains more functional groups capable of establishing polar interactions compared to HU. However, the thermodynamic parameters associated with the partitioning of both HU and Cyt in pluronic micellar media are found to be comparable. The enthalpy of partitioning of these drugs in both pluronic micelles is weakly exothermic and the partition process is predominantly entropically driven. The results suggest significant role of desolvation in the partitioning of drugs in the hydrophilic shell of pluronic micelles.

Like Cyt, Gem is also a deoxycytidine analogue in which hydrogen atoms at 2′ carbon on furanose ring have been replaced with fluorine atoms. Although Cyt and Gem have close structural similarity, the enthalpy of partitioning of Gem in pluronic micelles is observed to be 10 folds higher than that for Cyt and the enthalpy of partitioning is weakly endothermic. The partitioning coefficients are of the order of 10^4^ and 10^3^ in F127 and F68 micelles, respectively. The relatively higher hydrophobic nature of Gem due to fluorine substitution is accountable for the stronger partitioning of the latter in pluronic micelles than Cyt. Due to the enhanced hydrophobic character, Gem is able to interact with the hydrophobic PPO core in addition to the hydrophilic PEO shell which leads to stronger encapsulation.

**Fig. 9:**
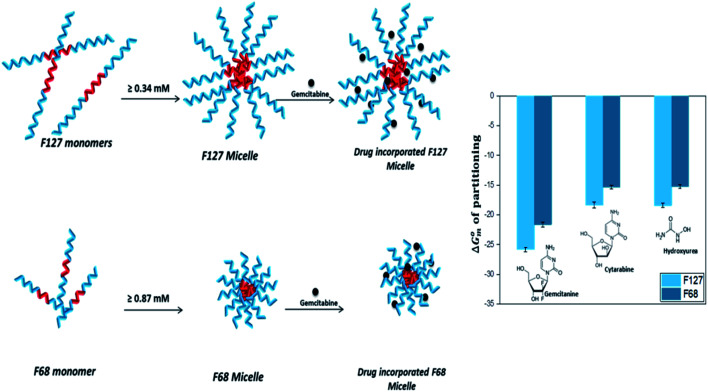
Schematic representation of incorporation of drugs in F127 and F68 micelles.

The incorporation of all the three drugs in F127 micelles is 10 fold stronger than that in F68 micelles. This can be attributed to relatively larger size of F127 micelles due to their greater block chain length. The average number of PPO units in F68 is almost half of that in F127. The steric hindrance due to the smaller size of the F68 micelles results in weaker partitioning. A schematic representation of incorporation of drugs in pluronic F127 and F68 micelles is shown in Fig. 9.

## 4 Conclusions

ITC helped to provide quantitative insights into partitioning of anticancer drugs cytarabine, gemcitabine, and hydroxyurea in pluronic F127 and F68 micelles. The partitioning of the drugs is observed to be weak to moderate with the values of partitioning constant ranging from 10^2^–10^4^ which is slightly temperature dependent. The results suggest that the two fluoro groups in gemcitabine lead to its stronger partitioning in both the micelles amongst the studied drugs. It is known that C–F groups impart more hydrophobicity compared to C–H groups.^42^ In general F127 micelles are observed to offer one order more partitioning compared to F68 micelles due to larger size as well as higher hydrophobic to hydrophilic components ratio in the former. Interestingly the studied drugs cytarabine, gemcitabine, and hydroxyurea did not show any binding profile on ITC, though fluorescence studies provided an affinity constant of the order 10^2^ for all these drugs. Therefore the entropic support drives the binding process. However, when drugs are delivered from pluronic micelles, well-defined binding profiles were observed on the ITC yielding affinity constant of the order of 10^4^ for these drugs and also strengthened binding evidenced by fluorescence measurements. These are important observations suggesting enhanced binding of drugs with serum albumin when delivered from F127 or F68 micelles irrespective of their structures. The CD and DSC results confirm that the integrity of binding sites on BSA is not significantly affected by pluronic micelles at the studied concentration. Such studies are important in identifying the functionalities required on self-assemblies as well as on the drugs to develop suitable drug delivery vehicles and also to assess the success of delivery without leading to major conformational changes to binding pockets on the target protein.

## Author contributions

Pooja Prasanthan: conceptualization, experimentation, data analysis, writing-original draft preparation. Nand Kishore: conceptualization, supervision, writing – review and editing, acquiring funds.

## Conflicts of interest

The authors declare no conflict of interest.

## Supplementary Material

RA-011-D1RA03770F-s001
