# Supplementary material for: Self-assemblies of pluronic micelles in partitioning of anticancer drugs and effectiveness of this system towards target protein
Source: RSC Adv. 2021 Jun 22;11(36):22057–69. doi: 10.1039/d1ra03770f (PMC9034178; doi:10.1039/d1ra03770f)
Supplement: RA-011-D1RA03770F-s001 [file RA-011-D1RA03770F-s001.pdf]

Electronic Supplementary Material (ESI) for RSC Advances.
This journal is © The Royal Society of Chemistry 2021

Supplementary Information

Self-assemblies of pluronic micelles in partitioning of anticancer drugs and effectiveness of this system towards target protein

Pooja Prasanthan, Nand Kishore*

Department of Chemistry, Indian Institute of Technology Bombay, Powai, Mumbai – 400 076, India.

*Corresponding author [Email: nandk@chem.iitb.ac.in]

List of Figures

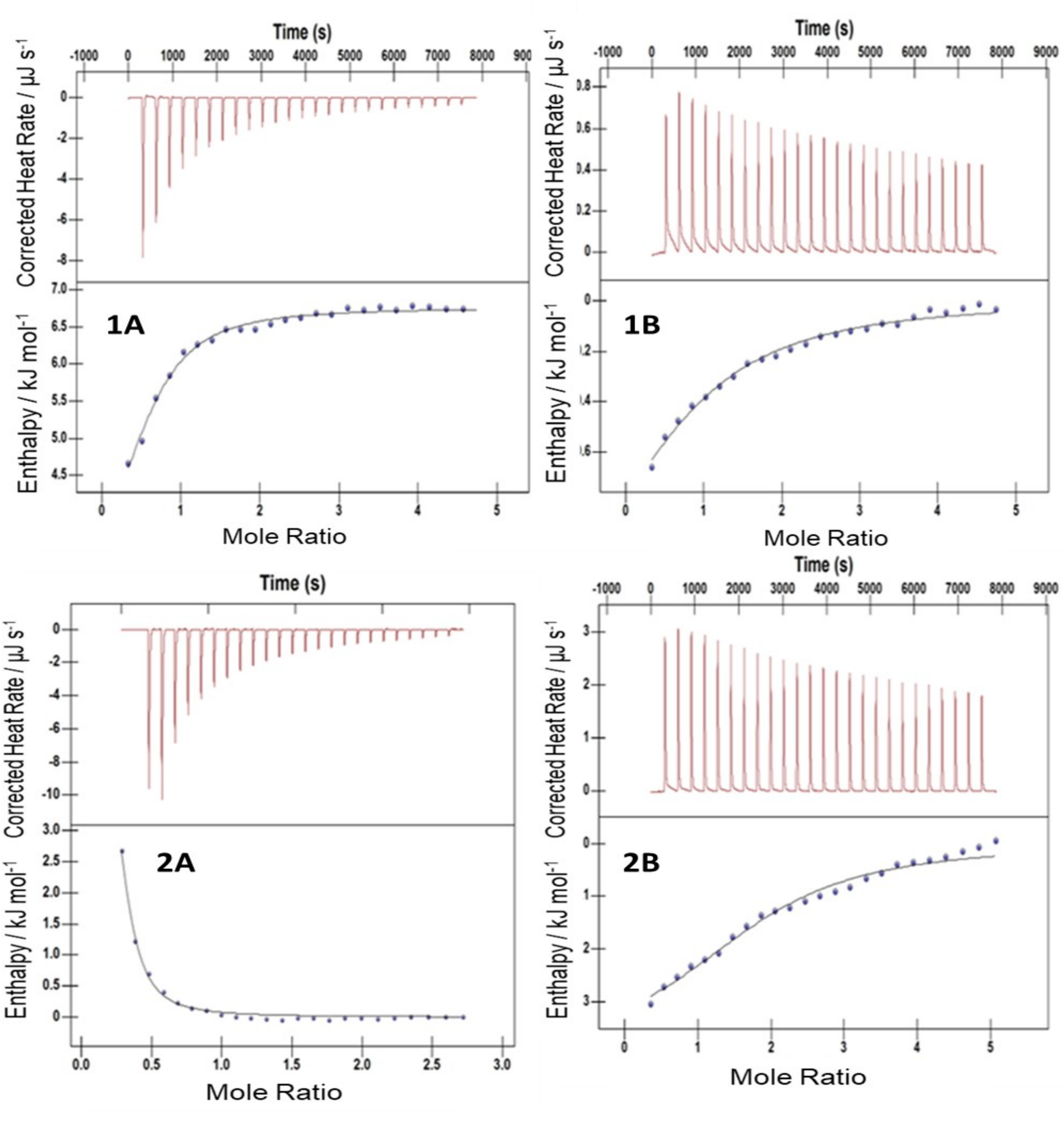

Fig S1 ITC profiles for titrations of (A) gemcitabine (B) hydroxyurea with (1) 1.1 mM F127 and (2) F68 micelles at 298.15 K

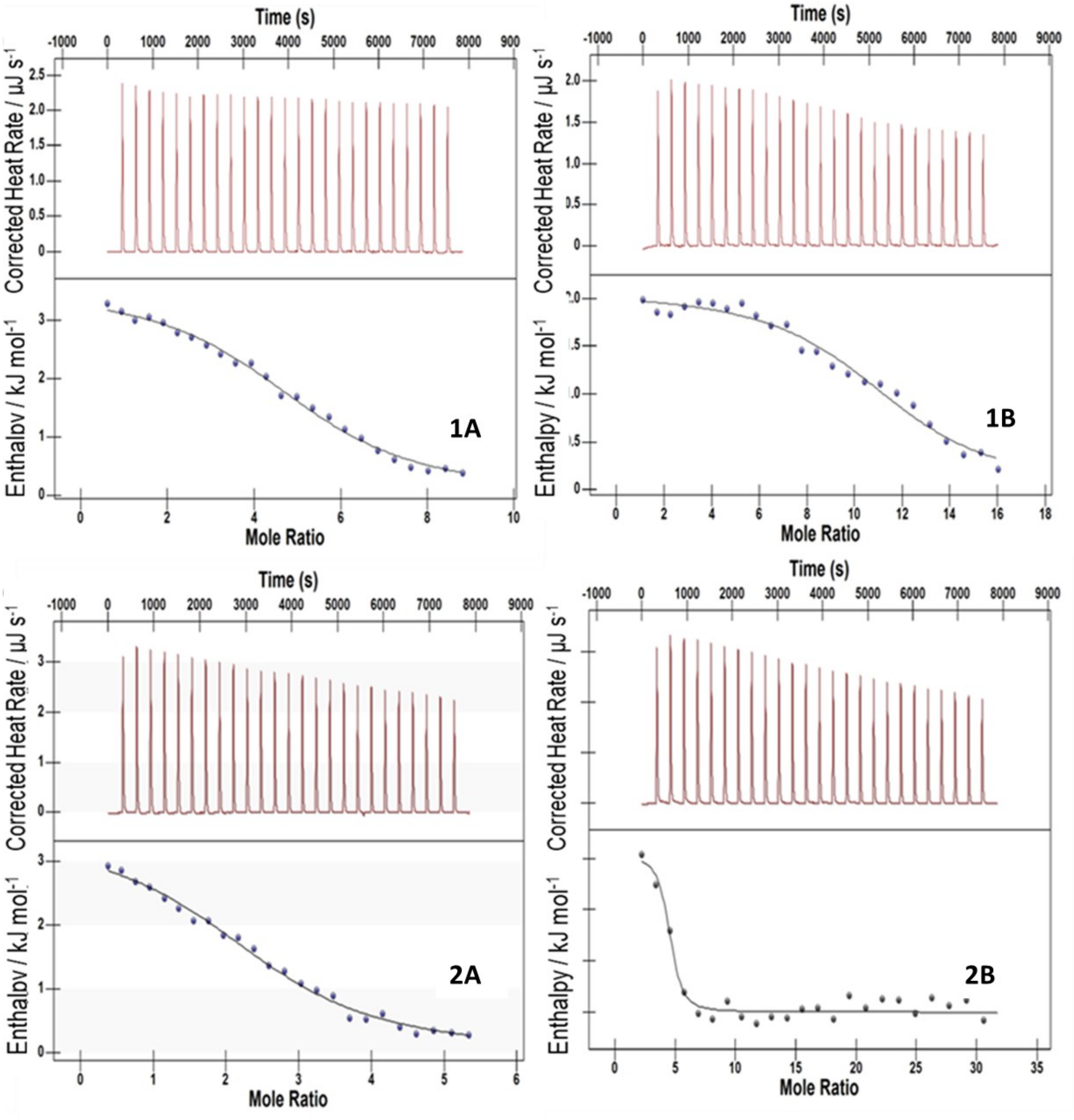

Fig S2 ITC profiles for the interaction of (A gemcitabine (B) hydroxyurea with BSA in the presence of (1) F127 and (2) F68 at 298.15 K

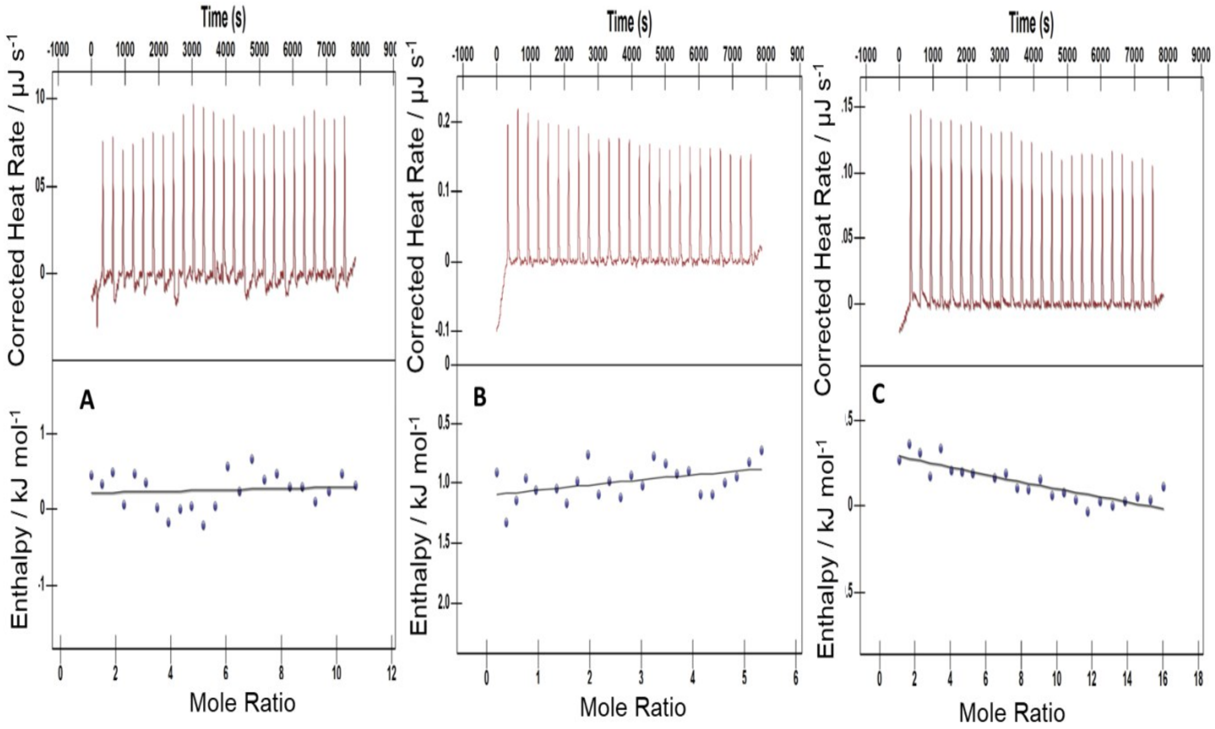

Fig. S3 The ITC profiles of interaction of (A) cytarabine, (B) gemcitabine, and (C) hydroxyurea with BSA at pH 7.4 and 298.15 K

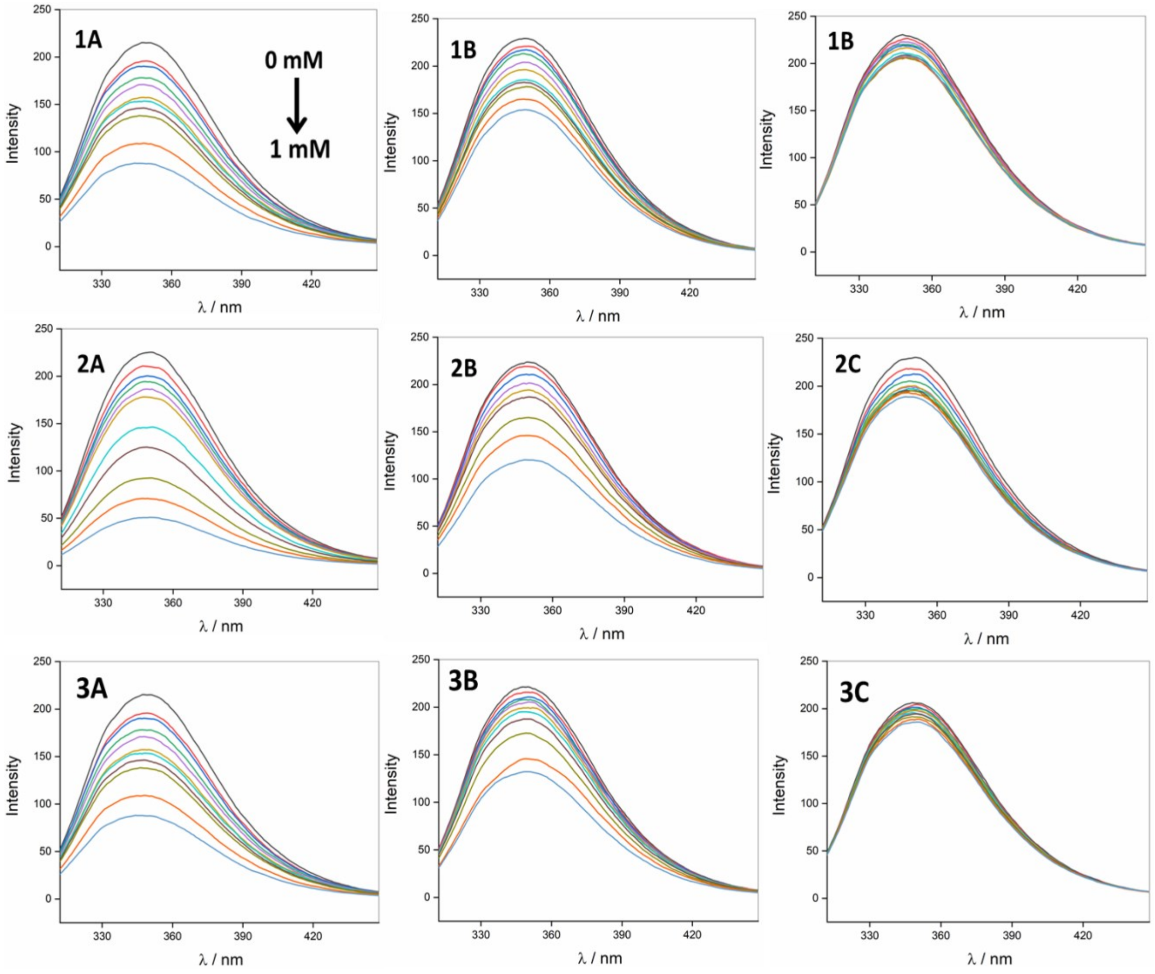

Fig. S4 Intrinsic fluorescence emission spectra of BSA with increasing concentrations of (A) cytarabine (B) gemcitabine and (C) hydroxyurea when released from (1) F127 and (2) F68 micellar media at pH 7.4 and 298.15 K.

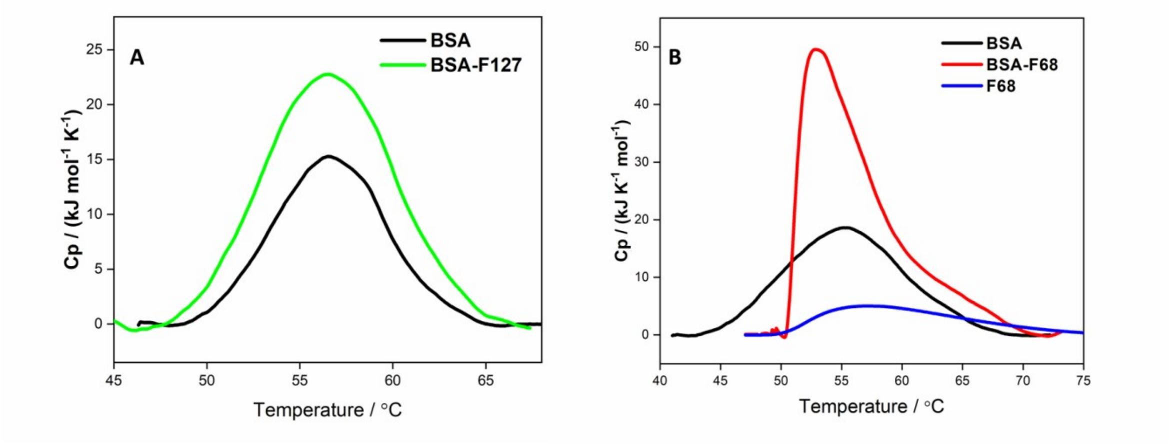

Fig. S5 DSC profiles of 3 mg ml^{-1} BSA in the absence and presence of (A) F127 and (B) F68
